# Nonverbal Skills Evolution in Children with Autism Spectrum Disorder One Year Post-Diagnosis

**DOI:** 10.3390/children11121520

**Published:** 2024-12-14

**Authors:** Maria Grazia Logrieco, Emma Annechini, Laura Casula, Silvia Guerrera, Mirco Fasolo, Stefano Vicari, Giovanni Valeri

**Affiliations:** 1 Department of Humanities, University of Foggia, Via Arpi 176, 71122 Foggia, Italy; maria.logrieco@unifg.it; 2 Department of Neuroscience, IRCCS Children’s Hospital Bambino Gesù, Piazza Sant’Onofrio, 4, 00165 Rome, Italy; laura.casula@opbg.net (L.C.); silvia.guerrera@opbg.net (S.G.); giovanni.valeri@opbg.net (G.V.); 3 Department of Neuroscience, Imaging and Clinical Sciences, University “G. d’Annunzio” Chieti-Pescara, Via dei Vestini 33, 66100 Chieti, Italy; mirco.fasolo@unich.it; 4 Department of Life Science and Public Health, Università Cattolica del Sacro Cuore, 00168 Rome, Italy; stefano.vicari@opbg.net

**Keywords:** autism spectrum disorder, gestures, intervention, preschoolers, nonverbal skills

## Abstract

**Background**: Gestural production, a crucial aspect of nonverbal communication, plays a key role in the development of verbal and socio-communicative skills. Delays in gestural development often impede verbal acquisition and social interaction in children with Autism Spectrum Disorder (ASD). Although various interventions for ASD focus on improving socio-communicative abilities, they consistently highlight the importance of integrating gestures to support overall communication development. This study aimed to investigate the progression of gestural production in preschoolers with ASD one year post-diagnosis, taking into account whether they had received interventions for ASD. **Method**: This study followed 76 Italian preschoolers with ASD, aged 2 to 4 years, who underwent three different types of interventions or no intervention at all. Data on gestural production were collected using the MCDI, a standardized parent-proxy report. **Results**: The results indicate that all groups, regardless of intervention type, experienced increased gesture production, suggesting that interventions, combined with factors like time, symptom severity, and learning differences unique to ASD, positively influence nonverbal communication. This improvement may be due to various factors. On one hand, joint attention and socio-communicative interactions drive progress, while on the other, children with ASD may benefit from learning through non-socially mediated linguistic material. **Conclusions**: These findings highlight the need to understand individual learning preferences and strategies for developing nonverbal communication skills in children with ASD. Identifying effective strategies early on can enhance both diagnosis and intervention planning, ensuring they are tailored to the specific developmental needs of each child.

## 1. Introduction

Autism Spectrum Disorder (ASD) is a complex and heterogeneous condition characterized by early difficulties in social interaction and mutual social communication, along with restricted and repetitive behaviors and interests [1,2]. ASD is a relatively common condition across the lifespan, affecting about 2% of the world’s population [3,4,5]. In Italy, it is estimated that 1 in 77 children has a diagnosis of ASD, with a higher prevalence among males. The male-to-female ratio is approximately 4/5:1 [6]. Research shows that children with ASD exhibit poorer and less varied communicative and social skills compared to typically developing children (TD) of the same age [7,8,9,10,11]. The most impaired socio-communicative areas include socio-emotional reciprocity and peer interest, difficulties in forming and maintaining social relationships and impairment in the use of nonverbal interactive behaviors, such as voice pragmatics, eye contact, facial expressions, and gestures [1]. Although autism presents with highly heterogeneous characteristics, many children with ASD show language skills significantly below what would be expected for their cognitive and/or developmental level [12,13]. Some studies explored the developmental trajectories of gesture and language in both ASD and TD children during their first year of life, finding no significant differences between the two groups, which included those with a high risk of ASD [14,15,16]. However, this trajectory appears to diverge in the second year of life for children with ASD, often characterized by a regression in communicative abilities [17]. This regression may affect gestural production, language skills, and social engagement [18]. Other studies have shown that autistic development is characterized not only by a lower quantity of gestural production [19,20,21,22,23,24,25,26] but also by a qualitative deficit in communicative gestures. This includes greater impairment of deictic gestures [27] and difficulties in mastering instrumental gestures [22]. Gordon and colleagues [28] studied the gestural production of children aged 13 to 15 months, with a follow-up at 20-24 months. They found that children who later received an autism diagnosis generally exhibited lower gestural production. During the follow-up evaluation, researchers observed that reduced gestural production was associated with greater impairment of social/communication skills.

### 1.1. Gesture–Language Association in Typical Development vs. Autism Spectrum Disorder

In developmental psychology, the association between gestures and language has been widely studied, with early gesture production identified as a strong predictor of future language development [29]. In a longitudinal study, Rowe and colleagues [29] considered 53 families and studied parent–child interactions, observing that gesture use at 14 months was a significant predictor of vocabulary size at 42 months of age. However, parental gestures were linked to the child’s use of gestures at 14 months, which in turn influenced their language development. The evolution of gestural skills in ASD is also influenced by their interactions. Some studies indicate that children with ASD acquire gestures through imitation during natural interactions [30,31]. Moreover, several studies have shown that despite delayed language and gesture production in children with ASD, the association between gesture and language remains significant [32]. In a study involving children at risk of ASD, Iverson and colleagues [33] examined various communicative strategies (vocalizations and gestures) and their integration for communicative purposes in both children at risk of ASD and TD children at 13 and 18 months of age. They found that the at-risk group exhibited lower levels of communicative behaviors at both ages compared to the TD group [33]. Similarly, Riva and colleagues [34] conducted a longitudinal study on the developmental trajectories of linguistic and gestural development in children at risk of ASD, with follow-ups at 12, 18, and 24 months of age. Consistent with previous studies [35,36], they found that expressive language levels were associated with gesture production at 12 and 18 months. Children with fewer gestural skills later experienced greater difficulties in language acquisition and appeared more impaired in socio-communicative terms [34]. This suggests that increasing gesture use and production in children at risk of ASD or those already diagnosed provides a crucial means of nonverbal communication, which is linked to enhanced communicative strategies, linguistic development, and improved social interaction skills [14]. Other studies involving children aged 2 to 4 show that gesture production in children with ASD is lower than in TD children, and this deficit significantly affects adaptive behavior and social abilities [37]. Additionally, deficits in gesture production at 36 months have an impact on vocabulary development one year later [24]. Difficulties in social communication and the use of gestures for communicative purposes, along with other significantly impaired competencies in autism, such as a lack of response to one’s name or difficulty in modulating eye contact, are often considered early warning signs for an ASD diagnosis and form the basis of many intervention strategies [28,38,39].

### 1.2. Early Intervention in Autism Spectrum Disorder

There is strong consensus in the literature regarding the importance of early intervention in ASD [40,41], and many support plans are designed to address the core symptoms of ASD, including linguistic skills and gesture training. Among the empirically validated and evidence-based treatments for ASD, psychosocial interventions, whether delivered by clinicians or parents, have shown efficacy in various areas [42,43]. Since there is no evidence supporting a single best intervention for all children with ASD [44,45], psychosocial interventions range from highly structured approaches focused on behavioral analysis (ABA) to naturalistic and developmental interventions (NDBIs) and teaching models (TEACCH) [46,47,48]. While none of these interventions focus exclusively on gestural production, each incorporates gestures and nonverbal communication for various purposes. The comprehensive individual approach based on ABA principles is a therapeutic intervention rooted in behaviorism [49]. In this type of intervention, the promotion of language, including gestures, is based on the need to make requests and is employed in structured one-to-one interactions with a therapist [6,50]. NDBIs are models that integrate both behavioral and developmental strategies. NDBIs, such as the Early Start Denver Model (ESDM) [48], emphasize the importance of encouraging spontaneous initiatives and flexible responses in children, particularly through the functional use of gestures for social communication and interaction [51]. Shared attention toward both objects and people, and imitation play a crucial role in learning and social integration, even prior to the development of language skills [31,48,52,53,54,55]. Moreover, upon receiving a diagnosis, most preschoolers with ASD typically undergo other nonspecific interventions for ASD, such as speech therapy and psychomotor therapy, commonly referred to as Therapies As Usual (TAU). Both interventions aim to enhance communication skills in preschoolers with ASD, incorporating the use of gestures. In particular, psychomotricity incorporates the use of gestures, working on motor skills related to the communicative difficulties of autism [56,57,58,59]. Speech therapy integrates gestures to enhance and support language development and social communication [24,60,61].

### 1.3. The Current Study

As highlighted in the brief literature review, the atypical development of gestural production in preschoolers with ASD significantly impacts several developmental goals, including verbal communication and socio-communicative skills. Gestures, as a key component of nonverbal communication, often serve as a precursor to spoken language, and delays or deviations in gestural development can hinder the acquisition of verbal skills. Furthermore, impaired gestural use may affect a child’s ability to engage in social interaction and learning. The recommended interventions for ASD address socio-communicative skill enhancement in various ways, but they all emphasize the importance of integrating gestures to support broader communication goals. Limited research has examined the evolution of gestural production in children participating in various interventions over the course of a year while also considering a control group of children who did not receive any intervention. Including a control group could offer valuable insights into the development of gestural production among preschoolers with ASD [62,63]. Therefore, the aim of the study was to explore the evolution of gestural production in preschoolers with ASD one year after diagnosis, considering whether the child had undergone an intervention for ASD. To our knowledge, no studies have specifically focused on the evolution of gesture production in the context of these interventions.

## 2. Materials and Methods

### 2.1. Participants

**Figure 1:**
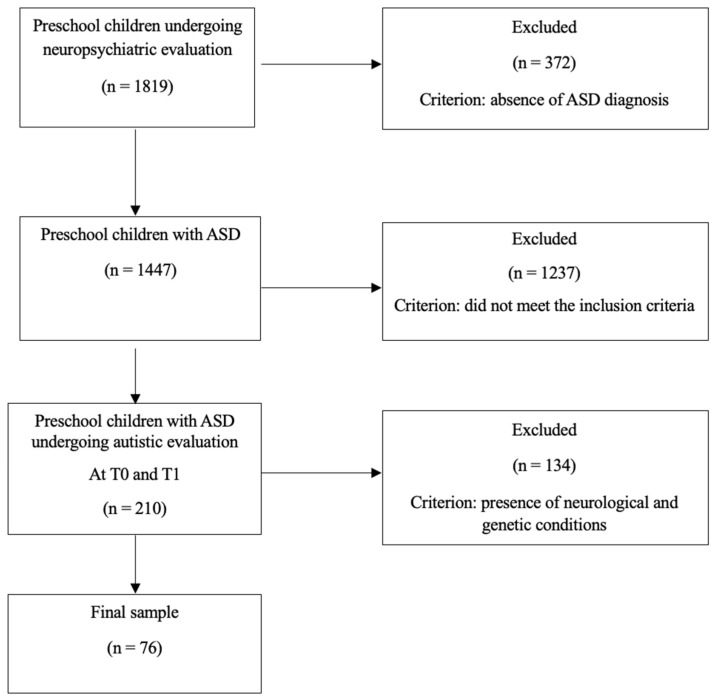
The workflow of this study.

This study utilized a longitudinal research design. Data were gathered retrospectively through a detailed review of patient records from the Child and Adolescent Neuropsychiatry Unit at a Children’s Hospital. The review included cases from 2019 to 2023 involving patients referred for neuropsychiatric evaluation based on a suspicion of ASD by a pediatrician, as well as follow-ups conducted one year after ASD diagnoses. Comprehensive assessments were conducted, including neuropsychiatric, psychopathological, and ASD symptom evaluations and assessments of adaptive and cognitive functioning. Parents were also asked about the types of interventions their child had undergone. The exclusion criteria were the existence of neurological conditions (e.g., epilepsy) or genetic conditions and a lack of follow-up after the initial diagnostic visit (one year after). This study adhered to the principles outlined in the Declaration of Helsinki and received approval from the local Ethics Committee (protocol code: 2423_OPBG_2021, approved on 27 October 2021). Of the 1819 preschool-aged children who underwent neuropsychiatric evaluation, 1447 received a diagnosis of ASD based on the DSM-5 criteria. Of these, 1237 did not complete the follow-up after the initial diagnostic visit (one year after). Two hundred and ten preschool-aged children with ASD received medical and developmental evaluations, which included diagnostic assessments of autism conducted by a team of child psychiatrists and psychologists, before (T_0_) and after intervention (T_1_). The final sample comprised 76 Italian preschoolers of both sexes with ASD that were between 2 and 4 years old at T_0_ (58 males; mean age in months at T_0_ = 32, SD = 1.1; 30 children with IQ < 70; 24 children with IQ between 70 and 85; 22 children with IQ > 85). Figure 1 provides a summary of this study’s workflow. The children were distributed among the groups as follows: 20 children performed ABA; 19 children received an NDBI; 19 children received TAU; and 18 children did not undergo interventions (Table 1). The average treatment duration per week ranged from 4 h for the TAU and NDBI groups to 8 h for the ABA group. Refer to Table 1 for descriptive statistics of the demographic information and clinical characteristics of the included participants.

### 2.2. Materials

#### 2.2.1. Gesture Assessment

The Primo Vocabolario del Bambino (PVB) questionnaire is the Italian version of the MacArthur–Bates Communicative Development Inventory (MB-CDI) [64,65]. These parental questionnaires assess the communication and language development of children aged 8 months to 36 months. The PVB has been widely used in research involving both typical and atypical Italian child populations [66]. It includes two forms: “PVB—Gesture and Words” and “PVB—Words and Sentences”. The authors [64] established normative data for the Italian MB-CDI in their manual “Words and Gestures and Words and Sentences”, which was recently updated [66]. The parents completed the “PVB—Gesture and Words” at T_0_ and T_1_ because the other versions of the MB-CDI do not take gestures into account. This version was the most appropriate for the developmental level of the children in our sample. Therefore, raw scores from the gesture scale were used.

#### 2.2.2. Autism Diagnostic Observation Schedule, Second Edition [ADOS-2] [67]

The ADOS-2 is the gold-standard instrument used for the assessment of ASD symptoms. It is a semi-structured, face-to-face evaluation that aims to directly assess communication and social interaction skills and the use of play or creativity in individuals who are suspected of having ASD [67]. It includes five different modules designed for individuals with different language abilities. The ADOS-2 is administered by trained clinicians, who derive a total score based on symptoms observed in the Social Affect (SA) and Restricted and Repetitive Behavior (RRB) domains. This assessment utilizes a combined total calibrated severity score (TOT CSS), which incorporates both the SA calibrated severity score (SA CSS) and the RRB calibrated severity score (RRB CSS). This assessment was conducted at both T_0_ and T_1_. The ADOS-2 exhibits high interrater and test–retest reliability, as well as strong predictive accuracy and specificity in differentiating ASD from non-spectrum conditions [68,69]. It has also been modified for use in different countries. For the present study, the reliability coefficients were αSA = 0.83, αRRB = 0.81, and αTOT = 0.81.

### 2.3. Additional Measures

#### Griffiths Mental Development Scales [GMDSs]

In this study, the participants’ cognitive or developmental levels were evaluated using the Global Developmental Quotient (GDQ), which was calculated using the Griffiths Mental Development Scales—Extended Revised 0–2–GMDS-ER 2–8. This tool measures children’s developmental progress across five domains for ages 0 to 8 years [70,71,72]. Each domain yields a separate developmental quotient, offering valuable insights into early developmental challenges. The overall GDQ is the average of these individual quotients. Children with a GDQ score below 70 were identified as having cognitive impairment, while those scoring 70 or above were regarded as unimpaired.

### 2.4. Plan of Analysis

Data analysis was conducted using R statistical software (2021 version) [73]. A Univariate analysis of Variance (ANOVA) was conducted to determine if there were differences in the ages of the children in the different groups at T_0_, PVB gesture production, ADOS II severity scores, and GMDS-ER scores. In addition, hierarchical linear models were used to examine the predictors of gesture production among the four intervention groups, taking into account the severity of the autistic symptoms and time. In particular, the following models were tested and compared: [1] Model 1, which included ADOS II scores and time as predictors of gesture production; [2] Model 2, which helped to identify the main effects of the factors ADOS II, time, and intervention on gesture production; and [3] Model 3, which examined whether the effect of timing on gesture production differed depending on the intervention type. To compare the models and select the one that received the most support, we used a comparative fit index—the Bayesian Information Criterion (BIC)—with lower values providing more support for a model compared to the previously tested model.

## 3. Results

**Figure 2:**
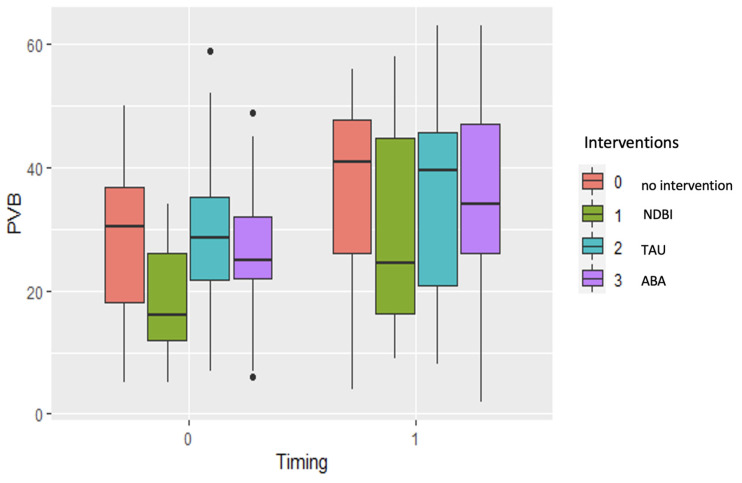
Box plot showing different intervention groups (no intervention, naturalistic and developmental behavioral interventions (NDBIs), applied behavioral analysis (ABA), and therapies as usual (TAU)). Gesture production was measured with PVB (on Y axis) at T_0_ and T_1_ (X axis).

Descriptive statistics for the GDQ, PVB, and ADOS scores at T_0_ and T_1_ are presented in Table 2. An ANOVA between all groups showed no significant effects for the differences in ages (F_(3,78)_ = 1.81; *p* = 0.15), PVB gesture production (F_(3,73)_ = 1.32; *p* = 0.27), ADOS II severity scores (F_(3,45)_ = 2.35; *p* = 0.85), and GMDS-ER scores (F_(3,62)_ = 1.41; *p* = 0.24) at T_0_ and for PVB gesture production at T_1_ (F_(3,73)_ = 0.351; *p* = 0.78). The ages of the children showed no significant correlations with PVB gesture production in either the pre-intervention phase or the post-intervention phase (pre, r = 0.035, *p* = 0.768; post, r = −0.31, *p* = 0.793). In all groups, there were significant increases in gestural production between T_0_ and T_1_: no intervention (t (17) = 8.36, *p* = 0.001); NDBI (t (18) = 3.74, *p* = 0.05); TAU (t (18) = 9.03, *p* = 0.001); ABA (t (19) = 9.90, *p* = 0.001). Regarding the tested models, several models were compared to assess the effects of different predictors on the outcome variable. The BIC was used as a measure of model fit, with lower values indicating better fit. The null model yielded a BIC of 1222. Introducing the interaction between ADOS II scores and timing resulted in a BIC of 1176. Including the main effects of ADOS II scores, timing, and intervention led to a BIC of 1174. Finally, a model incorporating the interaction between ADOS II scores, timing, and the type of intervention achieved the lowest BIC of 1170. These results suggest that the model including the interaction term provided the best fit for the data, as indicated by the lowest BIC value. To explore the change in gestural production over time, we created a box plot. The box plot shows the gesture production in the different intervention groups (NDBI, ABA, TAU, and no intervention) at T_0_ and T_1_. This graphical exploration of change over time shows that there were great increases in gestural production skills in all groups (NDBI, ABA, TAU, and no intervention). However, there was great variability in all groups, indicating the need to consider additional moderating conditions (Figure 2).

## 4. Discussion

This study aimed to investigate the development of gestural production in preschoolers with ASD one year after diagnosis, taking into account whether the child received intervention for ASD. In a sample of Italian preschoolers, three distinct groups underwent different types of interventions (NDBI, TAU, and ABA), while one group received no intervention. Few studies have considered the evolution of gestural production in children with ASD who have undergone different interventions for a year while also taking into account a control group consisting of children who have not undergone any intervention. The inclusion of a control group provides valuable insights into the development of gestural production in preschoolers with ASD [62,63]. The data indicate that all intervention groups, as well as the control group, experienced increases in gesture production. Additionally, the interaction of the temporal component, symptom severity, and the type of intervention influenced gesture production. Therefore, we can hypothesize that in all groups, despite the different intervention strategies and objectives, a positive effect on gesture production was observed [6,48,49]. In ABA interventions, gesture use is promoted through prompts and structured based on positive reinforcement. The primary use of gestures in ABA interventions is focused on the need to make requests, and they are used in structured one-to-one interactions with a therapist [6,42,49,50]. On the other hand, the NDBI model encourages the creation of opportunities for children to respond spontaneously and flexibly, including the functional use of gestures for social communication and interactional patterns [48].

### 4.1. Shared Attention and Gesture Production

Gesture production is recognized as a critical component that characterizes and promotes social and communication skills and is therefore encouraged within interactive spaces. Gestures are promoted through adult imitation and shared attention, which are essential for developing nonverbal skills and for learning, social integration, and other developmental outcomes, even during the prelinguistic stage [31,48,52,54,55]. In our study, gestural production increased after one year in both the group that underwent ABA therapy and the NDBI group, and the data align with previous studies conducted by other authors in recent years [74,75,76]. As a matter of fact, both interventions focus on enhancing nonverbal communication skills. For both groups, the development of gesture production is influenced by social interactions, particularly through the presence of shared attention, which facilitates the learning and use of gestural communication [42,48,49]. According to the literature, the use and comprehension of gestures, which emerge as development progresses, originate within interactions, and gestures have stable semantic contents that remain unchanged across different communicative situations [77,78,79]. Therefore, child–adult interaction plays a fundamental and crucial role in interpreting a child’s gestures within a joint attention space and in promoting their use until the vocal modality eventually prevails over the gestural one during development [33]. The same strategies that may lead to increases in nonverbal skills, such as the implementation of joint attention and socio-communicative interactions, are also employed in TAU. The models discussed above focus on the generalizability of the relationship between gestural production and language skills across typical and autistic development [17,32]. Thus, according to this explanation, the children who did not receive interventions may have experienced increases in gesture production due to interactions in everyday environments [42,48,49,78]. In addition, children of this age can also attend kindergarten, which influences their development, particularly in terms of social competencies [80]. Interactions with peers, both in groups and in one-to-one relationships, promote language development and enable children to engage in meaningful interactions and communicate [81].

### 4.2. A New Perspective on Language Acquisition

Recently, some researchers have proposed a different hypothesis that could provide additional insights into the results observed in our study. According to Mottron and colleagues [82], the mechanism of language acquisition in some children with ASD may differ from typical development in the extent to which it depends on joint attention skills [82]. In particular, different studies found that for some children with ASD, interest in linguistic input is not driven by communicative intent but often by an early and intense interest in written material. This suggests that some children with ASD are precociously engaged in learning from non-socially mediated linguistic material [12,83,84]. Thus, the increase in nonverbal skills may be due to the implementation of joint attention and socio-communicative interactions or a different learning mechanism for communication skills characteristic of ASD, as indicated by Mottron’s studies [32,82,85]. It is essential to understand each child’s preferred strategy for learning nonverbal communication skills, given their critical role in achieving various developmental goals [14,29,32,86,87]. By exploring which strategies children use and prefer for learning nonverbal communication early on, we can enhance both diagnosis and the effective structuring of interventions, aiming to respect the unique characteristics of each individual with ASD.

### 4.3. Limits and Future Directions

This study has several potential limitations. One notable constraint is the limited sample size of the children with ASD, which could impact the generalizability of the results to the broader population of preschool-aged children with ASD. Second, our study focused solely on the outcomes of preschool-aged children with ASD over a brief period. However, we believe that our findings underscore the importance of the preschool years as a critical period for the development of communicative skills. Furthermore, our findings focused solely on the developmental impact of the quantity of gesture production, without taking into account the quality of the gestures. These issues, along with other possible confounding variables, should be considered in future research. Another limitation is the reliance on parent-proxy reports to assess gestural skills. Although the PVB is filled out independently by parents, it is commonly utilized in both research and clinical settings to document and track progress over time [86,88]. Adding direct observation by a clinician could help mitigate these issues by providing a more objective and comprehensive assessment of gestural abilities. Moreover, the examination of changes over time revealed that all groups experienced significant increases in gestural production skills and variability. This suggests a need to consider additional moderating factors. Furthermore, we did not collect data on whether the children attended kindergarten. As we mentioned in the discussion, this could be an additional factor supporting the gain of gesture use in children with ASD [80,81]. Although there are limitations, this study has notable strengths, including the diversity and representativeness of the samples, which encompassed individuals across all levels of cognitive functioning, as well as the inclusion of a control group. Few studies include a control group, which is essential for fully understanding the physiological evolution of skills in preschoolers with ASD, or take into account groups that have undergone different interventions [62,63]. Future studies should be randomized controlled trials that compare different evidence-based interventions and control groups regarding their effectiveness in improving nonverbal communication.

## 5. Conclusions

In conclusion, our study explored the evolution of gestural production in children with ASD one year after diagnosis. These children had undergone various interventions or none at all during the year. The results showed increases in gestural production across all groups. Additionally, no intervention had a predictive effect on the outcome after one year. This may have been due to the strong interactive components of all the interventions or the different learning mechanism for communication skills characteristic of ASD. Early identification of ASD allows for timely intervention with targeted measures that also seek to improve nonverbal communication skills. Starting these interventions early for children at risk of ASD could be beneficial not only for enhancing language development but also for providing opportunities to monitor other developmental aspects for signs of ASD [28,86]. Additionally, it seems necessary to deepen our research on learning strategies for nonverbal communication, considering and respecting the unique characteristics of ASD and each autistic child.

## Figures and Tables

**Table 1:** The demographic characteristics of the sample. Intervention groups: applied behavioral analysis (ABA), naturalistic and developmental behavioral interventions (NDBIs), therapies as usual (TAU), and no intervention.

Intervention Groups	Number of Children	Age at T_0_, Mean (SD)
ABA	20 (6 Females)	33 (1.19)
NDBI	19 (2 Females)	30 (2.11)
TAU	19 (3 Females)	30 (2.35)
No intervention	18 (7 Females)	29 (3.71)

**Table 2:** Descriptive statistics of GMDS-ER (Griffiths Mental Developmental Scales—Extended Revised; Primo Vocabolario del Bambino (PVB); and Autism Diagnostic Observation Schedule, Second Edition (ADOS II) scores at T_0_ and T_1_. Number of children for every ADOS module.

Measure	T_0_ Mean (SD)		T_1_ Mean (SD)	
*Griffiths Mental Development Scales—Extended Revised*				
Global Developmental Quotient	69.45 (16.96)		67.19 (19.36)	
*PVB questionnaire*				
Understanding sentences	13.75 (9.09)		18.62 (8.82)	
Understanding words	92.59 (94.58)		189.69 (135.39)	
Word production	20.37 (50.78)		85.27 (120.86)	
Gesture production	26.88 (12.53)		36.38 (20.52)	
*ADOS II*				
		N		N
Toddler	5.55 (1.96)	37	3.86 (2.26)	13
Module 1	6.39 (1.28)	39	6.46 (1.51)	54
Module 2			5.88 (0.91)	9

## Data Availability

The dataset presented in this article is not readily available because it includes sensitive information about minors with developmental vulnerabilities. Requests to access the dataset should be directed to the corresponding authors.
